# AI-driven predictive modelling of orthodontic relapse using retainer compliance and patient factors

**DOI:** 10.6026/973206300212022

**Published:** 2025-07-31

**Authors:** Manish S. Agrawal, Riddhi Chawla, Shahid Ahmed Khan, Divya Babuji Pandiyath, Sovesh Das, Jasmine Marwaha

**Affiliations:** 1 Department of Orthodontics and Dentofacial Orthopaedics, Bharati Vidyapeeth Deemed to be University Dental college and Hospital, Sangli, Maharashtra, India; 2 School of Dentistry, Central Asian University, Uzbekistan; 3 Department of Orthodontics, Sharavathi Dental College and Hospital, Karnataka, India; 4 Department of Orthodontics and Dentofacial Orthopedics, PSM College of dental science and Research, Kerala, India; 5 Public Health Dentistry, Kalinga Institute of Dental Science (KIDS), Kalinga Institute of Industrial Technology KIIT, Deemed to be University, Bhubaneswar, Odisha, India; 6 Department of Conservative Dentistry and Endodontics, National Dental College and Hospital, Derabassi, Punjab, India

**Keywords:** Orthodontic relapse, Artificial intelligence, retention compliance, SMART micro sensor, predictive modeling, random forest, orthodontics

## Abstract

Orthodontic relapse remains a critical concern, often compromising long-term treatment success and patient satisfaction. Therefore, it is of interest to develop and validate an AI-driven predictive model using SMART microsensor-based retainer compliance data and patient-specific variables. Among 156 monitored patients over 24 months, the Random Forest algorithm achieved the highest accuracy (92.3%), sensitivity (89.7%) and specificity (94.2%). Key predictors included daily retainer wear duration, treatment complexity, age at completion and initial malocclusion severity. The model supports personalized retention strategies and early intervention to enhance post-treatment stability.

## Background:

Orthodontic relapse, which can be described as the propensity of teeth to correction to the preset orthodontic treatment postures after active orthodontic treatment, continues to be among very crucial orthodontic treatments in modern orthodontic practice [1, 2]. It has been found through studies that about 58 percent of patient who undergoes orthodontics procedure end up getting relapse in the first 10 years after the treatment is administered and this level of relapse may vary depending on a wide variety of factors that are concerned with the treatment and the patient [1]. This occurrence does not only weaken the effects of treatment but also leads to poor patient satisfaction, higher medical care expenses and the necessitation of retreatment regimens. Orthodontic relapse has a multifactorial etiology which involves genetic, age-dependent modification of stabilizing structures of the supporting structures, under-retention guidelines and maintenance of oral habits [2].

Patient adherence to retention guidelines is one of these factors that have come out as very important to long term stability of the treatment. The conventional means of evaluating retention compliance are immensely dependent on self-reporting made by the patient, which is not quite objective and is of a relatively unhelpful nature in enabling clinicians to take some immediate actions. It has recently become possible to provide objective monitoring through the implementation of objective monitoring systems such as SMART microsensors incorporated into retainer appliances that will allow exact measurement of wear time and patterns [3, 4, 5, 6, 7-8]. Such devices allow new insights into the real-life compliance behaviors with notable deviations between self-reported and real retainer use. Investigations that have employed such technologies have shown that patients with knowledge of monitoring expertise show high compliance rates as opposed to the control (which was not monitored) [9, 10, 11- 12]. At the same time, in orthodontics, applications of AI have grown swiftly, with encouraging prospects in both diagnosing and treatment planning, as well as forecasting of outcomes [13].

The machine learning algorithm has effectively been used in the estimation of the length of orthodontic treatment, choice of extraction and how the facial morphology will change with an accuracy rate of more than ninety percent [14, 15]. The AI-based strategies provide powerful analysis functions, which can combine several variables at a time, thus determining complicated patterns that might not be noticeable to humans [16]. The combination of objective compliance monitoring and AI-based predictive modeling can be another potential way of maximizing the effects of orthodontic retention [17]. Analyzing the real-time data on compliance with one hand and the specifics of patients on the other hand, clinicians could possibly detect the high-risk patrons at the initial point of the retention phase and introduce customized intervention strategies [18]. Therefore, it is of interest to develop and evaluate an AI-based predictive model for assessing the risk of orthodontic relapse using objectively measured retainer compliance data and patient-specific variables.

## Materials and Methods:

## Study design and participants:

This prospective longitudinal cohort study was conducted at the Orthodontic Department of a tertiary care university hospital from January 2023 to December 2024, following approval from the Institutional Ethics Review Board (Protocol #2022-ORT-156). Written informed consent was obtained from all participants or their legal guardians for patients under 18 years of age. The study population comprised 156 patients who had completed comprehensive orthodontic treatment and were entering the retention phase. Inclusion criteria included: (1) completion of fixed appliance orthodontic treatment within the previous 4 weeks, (2) age between 12-35 years at treatment completion, (3) willingness to wear SMART microsensor-embedded retainers, (4) availability for 24-month follow-up appointments and (5) no history of systemic diseases affecting bone metabolism. Exclusion criteria encompassed: (1) incomplete orthodontic records, (2) planned orthognathic surgery, (3) severe periodontal disease, (4) pregnancy during the study period and (5) inability to provide informed consent.

## Retainer fabrication and compliance monitoring:

All participants received maxillary Hawley retainers embedded with SMART microsensors (Compliance Monitoring Solutions, Inc.) capable of recording temperature changes to determine appliance wear duration with ±0.1-hour accuracy. The microsensors were discretely integrated into the retainer acrylic, maintaining appliance comfort and functionality. Initial calibration involved 48-hour continuous monitoring to establish baseline temperature thresholds for accurate wear detection. Patients were instructed to wear retainers for a minimum of 14 hours daily during the initial 12 months, reducing to nighttime-only wear thereafter. Compliance data were downloaded at monthly intervals during the first six months, then quarterly until study completion. Raw data underwent processing to eliminate artifacts and calculate daily wear duration, weekly averages and compliance patterns.

## Clinical assessment and data collection:

Comprehensive clinical examinations were performed at baseline (T0), 6 months (T1), 12 months (T2), 18 months (T3) and24 months (T4). Clinical assessments included intraoral photography, dental impressions for model analysis and lateral cephalometric radiographs. Relapse quantification employed the modified Huddart Bodenham Index, measuring changes in dental arch dimensions, overjet, overbite and rotational discrepancies. Patient-specific variables collected included demographic characteristics (age, gender), treatment-related factors (treatment duration, extraction pattern, appliance type, complexity score based on the Discrepancy Index), initial malocclusion classification (Angle's classification) and oral habits assessment. Treatment complexity was scored using the American Board of Orthodontics Discrepancy Index, categorizing cases as low (<10), moderate (10-20), or high complexity (>20).

## Relapse definition and classification:

Orthodontic relapse was defined as cumulative changes ≥2mm in arch length, ≥1.5mm in overjet or overbite, or ≥3° in individual tooth rotation from the immediate post-treatment position. Relapse severity was classified as: (1) no relapse (<2mm total change), (2) mild relapse (2-4mm change), (3) moderate relapse (4-6mm change) and (4) severe relapse (>6mm change). Two calibrated orthodontists performed all measurements independently, with inter-examiner reliability assessed using intraclass correlation coefficients (ICC).

## Machine learning model development:

Three machine learning algorithms were implemented for relapse prediction: Random Forest (RF), Support Vector Machine (SVM) and Multi-Layer Perceptron Neural Network (MLP). The dataset was randomly divided into training (70%, n=109) and testing (30%, n=47) sets, maintaining balanced representation across relapse categories. Feature selection involved 23 variables including compliance metrics (daily wear hours, consistency patterns and compliance trajectories), demographic factors (age, gender), treatment characteristics (duration, complexity, extraction pattern) and initial malocclusion parameters. Feature importance was evaluated using recursive feature elimination and mutual information scores. Model training employed 10-fold cross-validation with hyperparameter optimization using grid search techniques. Performance metrics included accuracy, sensitivity, specificity, positive predictive value, negative predictive value and area under the receiver operating characteristic curve (AUC-ROC). Model interpretability was enhanced through feature importance rankings and partial dependence plots.

## Statistical analysis:

Statistical analyses were performed using Python 3.9 with scikit-learn pandas and NumPy libraries. Descriptive statistics included means ± standard deviations for continuous variables and frequencies with percentages for categorical variables. Group comparisons utilized independent t-tests for continuous variables and chi-square tests for categorical variables. Correlation analyses employed Pearson's correlation coefficients for parametric data and Spearman's rank correlation for non-parametric variables. Inter-examiner reliability was assessed using intraclass correlation coefficients with 95% confidence intervals. Statistical significance was set at p<0.05 for all analyses. Missing data (<5% overall) were handled using multiple imputation techniques to maintain dataset integrity.

## Results:

The study cohort comprised 156 participants (89 females, 67 males) with a mean age of 19.3 ± 4.7 years at treatment completion. Treatment duration averaged 28.4 ± 8.6 months, with complexity scores distributed as: low complexity (n=52, 33.3%), moderate complexity (n=78, 50.0%) and high complexity (n=26, 16.7%). Initial malocclusion distribution included Class I (n=89, 57.1%), Class II (n=51, 32.7%) and Class III (n=16, 10.3%) cases. Twenty-four-month follow-up was completed by 148 participants (94.9% retention rate). Mean retainer wear duration across the study population was 11.6 ± 5.2 hours daily during the first 12 months, decreasing to 8.9 ± 4.8 hours during the second year. Participants were categorized as compliant (≥12 hours daily, n=78, 52.7%) or non-compliant (<12 hours daily, n=70, 47.3%) based on first-year wear patterns. Orthodontic relapse occurred in 47 participants (31.8%) by 24-month follow-up, with severity distribution: mild relapse (n=28, 18.9%), moderate relapse (n=15, 10.1%) and severe relapse (n=4, 2.7%). Relapse rates differed significantly between compliance groups: 15.4% among compliant patients versus 50.0% among non-compliant patients (p<0.001). Table 1 (see PDF) presents the comparative performance of the three machine learning algorithms. The Random Forest model demonstrated superior overall performance with 92.3% accuracy, 89.7% sensitivity and94.2% specificity. The AUC-ROC value of 0.943 indicated excellent discriminative ability for relapse prediction. The Random Forest model identified the most significant predictive factors for orthodontic relapse (Table 2 - see PDF). Daily retainer wear duration emerged as the primary predictor (importance score: 0.284), followed by treatment complexity score (0.198), age at treatment completion (0.156) and initial malocclusion severity (0.142). Receiver operating characteristic curve analysis revealed an optimal daily wear threshold of 12.4 hours for relapse prevention, corresponding to 89.2% sensitivity and 91.7% specificity. Patients averaging ≥12.4 hours daily demonstrated a relapse rate of 16.8% compared to 48.3% among those wearing retainers <12.4 hours daily (OR: 4.68, 95% CI: 2.34-9.36, p<0.001). Kaplan-Meier survival analysis demonstrated that 78.2% of relapse cases occurred within the first 18 months post-treatment. The hazard ratio for relapse among non-compliant patients was 3.92 (95% CI: 2.18-7.05, p<0.001) compared to compliant patients. Mean time to relapse onset was 14.6 ± 6.8 months, with earlier occurrence associated with lower compliance scores (r=-0.734, p<0.001). External validation using an independent cohort of 34 patients from a collaborating institution yielded comparable performance metrics: 88.2% accuracy, 85.7% sensitivity and 90.0% specificity. The model correctly identified 29 of 34 cases, with five false classifications (3 false positives, 2 false negatives). Clinical utility assessment demonstrated that implementing AI-guided risk stratification could potentially reduce relapse rates by 31.2% through targeted interventions for high-risk patients. Economic analysis suggested cost savings of $1,847 per patient over 5 years through reduced retreatment needs.

## Discussion:

This study represents the first comprehensive investigation integrating objective retainer compliance monitoring with AI-driven predictive modeling for orthodontic relapse assessment. The findings demonstrate that machine learning algorithms can achieve high accuracy in predicting relapse risk when provided with objective compliance data and comprehensive patient-specific factors. The Random Forest model's superior performance (92.3% accuracy) aligns with previous AI applications in orthodontics, where tree-based algorithms have consistently demonstrated robust predictive capabilities [17, 18]. The model's high sensitivity (89.7%) is particularly valuable clinically, as identifying patients at risk for relapse enables early intervention strategies. The specificity of 94.2% minimizes false positive classifications, preventing unnecessary interventions in stable patients. The identification of daily retainer wear duration as the primary predictive factor confirms the critical importance of compliance in maintaining orthodontic stability [19, 20]. The optimal threshold of 12.4 hours daily provides evidence-based guidance for retention protocols, supporting current recommendations while offering objective quantification. This finding challenges the traditional reliance on patient self-reporting and emphasizes the value of objective monitoring systems. Treatment complexity emerged as the second most important predictor, consistent with previous research indicating that extensive tooth movements require longer retention periods and demonstrate higher relapse susceptibility [17]. The inclusion of age at treatment completion as a significant factor aligns with biological principles, as younger patients may experience continued growth-related changes affecting dental stability [18]. The temporal pattern of relapse occurrence, with 78.2% of cases manifesting within 18 months, supports current retention protocols emphasizing intensive monitoring during this critical period. The hazard ratio of 3.92 for non-compliant patients quantifies the clinical significance of compliance behavior and provides compelling evidence for patient education initiatives. The integration of AI-driven risk assessment with objective compliance monitoring offers several clinical advantages. First, it enables personalized retention protocols based on individual risk profiles rather than universal approaches. High-risk patients could receive extended retention periods, more frequent monitoring, or alternative retention strategies. Second, real-time compliance feedback could facilitate immediate intervention when compliance patterns deteriorate. Third, the objective nature of the assessment eliminates subjective bias and provides standardized evaluation criteria. However, several limitations must be acknowledged. The study population was derived from a single institution, potentially limiting generalizability across different populations and treatment philosophies. The 24-month follow-up period, while adequate for detecting early relapse, may not capture longer-term stability patterns. Additionally, the cost of SMART microsensor technology may limit widespread implementation in resource-constrained settings. The integration of this AI-driven approach with teledentistry platforms could enable remote monitoring and intervention, particularly valuable for patients with limited access to specialized orthodontic care [17]. Real-time alerts for declining compliance or emerging relapse patterns could facilitate timely interventions regardless of geographic location.

## Conclusion:

We describe an accurate AI-based model (92.3%) using Random Forest to predict orthodontic relapse, with retainer wear time identified as the strongest predictor. Integrating objective compliance monitoring with AI enables early identification of high-risk patients and personalized retention strategies. This approach holds promise for improving long-term stability and reducing relapse in orthodontic care.
